# A broad spectrum anti-bacterial peptide with an adjunct potential for tuberculosis chemotherapy

**DOI:** 10.1038/s41598-021-83755-3

**Published:** 2021-02-18

**Authors:** Komal Umashankar Rao, Domhnall Iain Henderson, Nitya Krishnan, Manoj Puthia, Izabela Glegola-Madejska, Lena Brive, Fanny Bjarnemark, Anna Millqvist Fureby, Karin Hjort, Dan I. Andersson, Erik Tenland, Erik Sturegård, Brian D. Robertson, Gabriela Godaly

**Affiliations:** 1 Department of Microbiology, Immunology and Glycobiology, Inst. Laboratory Medicine, Lund University, Lund, Sweden; 2 Department of Infectious Disease, MRC Centre for Molecular Bacteriology and Infection, Imperial College, London, UK; 3 Department of Dermatology and Venereology, Inst. Clinical Sciences, Lund University, Lund, Sweden; 4 Bioscience and Material/Chemistry, Research Institutes of Sweden, Borås, Sweden; 5 Bioeconomy and Health/Chemical Process and Pharmaceutical Development, Research Institutes of Sweden, Stockholm, Sweden; 6 Department of Clinical Microbiology, Inst. Translational Medicine, Lund University, Malmö, Sweden; 7 Department of Medical Biochemistry and Microbiology, Uppsala University, Uppsala, Sweden

**Keywords:** Tuberculosis, Drug discovery

## Abstract

Alternative ways to prevent and treat infectious diseases are needed. Previously, we identified a fungal peptide, NZX, that was comparable to rifampicin in lowering *M. tuberculosis* load in a murine tuberculosis (TB) infection model. Here we assessed the potential synergy between this cationic host defence peptide (CHDP) and the current TB drugs and analysed its pharmacokinetics. We found additive effect of this peptide with isoniazid and ethambutol and confirmed these results with ethambutol in a murine TB-model. In vivo, the peptide remained stable in circulation and preserved lung structure better than ethambutol alone. Antibiotic resistance studies did not induce mutants with reduced susceptibility to the peptide. We further observed that this peptide was effective against nontuberculous mycobacteria (NTM), such as *M. avium* and *M. abscessus*, and several Gram-positive bacteria, including methicillin-resistant *Staphylococcus aureus*. In conclusion, the presented data supports a role for this CHDP in the treatment of drug resistant organisms.

## Introduction

The dramatic increase in antimicrobial resistance (AMR) makes infectious diseases a global medical challenge. The WHO recently identified the most important resistant bacteria for which there is an urgent need for new treatments, comprising the Gram-positive pathogens *Streptococcus pneumoniae* and *Staphylococcus aureus*, as well as *Mycobacterium tuberculosis*^1^. The total deaths from methicillin-resistant *Staphylococcus aureus* (MRSA) are now comparable to those caused by HIV, and it is estimated that by the year 2050, at least 10 million people will die annually due to AMR^2^. Within the genus *Mycobacterium*, comprising the *M. tuberculosis* complex and the large group of non-tuberculous mycobacteria (NTM), the resistance to drugs constitutes a formidable obstacle to effective care and global TB prevention^3^. Annually about 8.4 million people are diagnosed with TB, which kills an estimated 1.5 million people every year. NTMs cause mostly pulmonary diseases with similar pathology to TB, that can become chronic and affect quality of life for patients, although with less mortality^4^. Mycobacterial infections are very difficult to treat as this pathogen contains subpopulations of antibiotic persistent bacteria which can exist as drug**-**tolerant latent organism**s** in some patients that can reactivate to cause active transmissible infection. Current standard treatments for drug-sensitive TB and NTMs are based on multi-drug cocktails which last for several months. This time-consuming treatment-regimen is necessary to prevent the selection of drug-resistant mutants, which may arise during the course of treatment. However, each year more than half a million people are diagnosed with rifampicin-resistant TB. Bedaquiline and delamanid were approved for treatment of drug-resistant TB between 2012 and 2014 representing a critical milestone in anti-TB drug discovery, but clinical resistance to these compounds was reported less than three years after their introduction^5^. The NTMs also display a high degree of endogenous AMR and present an increasing clinical concern worldwide. Diverse bacterial growth rates are known to impact on the success of antibiotic therapy^6^. Fast growing NTM, such as *M. abscessus* spp., are for example, regularly resistant to available first-line TB drugs^7^. Further research for new drug targets and improved treatment strategies are thus urgently needed.

In recent years, cationic host defence peptide**s** (CHDP) have gained interest as potential novel drugs. These molecules are oligopeptides produced as part of the host defense and divided into cathelicidins and defensins. We previously identified a peptide, NZX, originating from the fungus *Pseudoplectania nigrella*^8^. The peptide’s antimicrobial capacity was analyzed against clinical isolates of *M. tuberculosis* and multi-drug resistant (MDR) *M. tuberculosis*, and in murine TB infection models, where NZX significantly lowered the bacterial load to levels comparable with rifampicin treatment^9, 10^. In this study, we analyzed the pattern of resistance to NZX, serum elimination half-life and its interaction with current TB antibiotics. We also assayed the peptide’s antimicrobial activity against a broad range of mycobacterial spp. and Gram-positive bacteria. We found that NZX displays substantial broad-spectrum antimicrobial efficacy, is synergistic with ethambutol in a murine TB-infection model and displays no antagonism with standard TB drugs.

## Results

### Antimicrobial activities of NZX against bacteria

**Figure 1:**
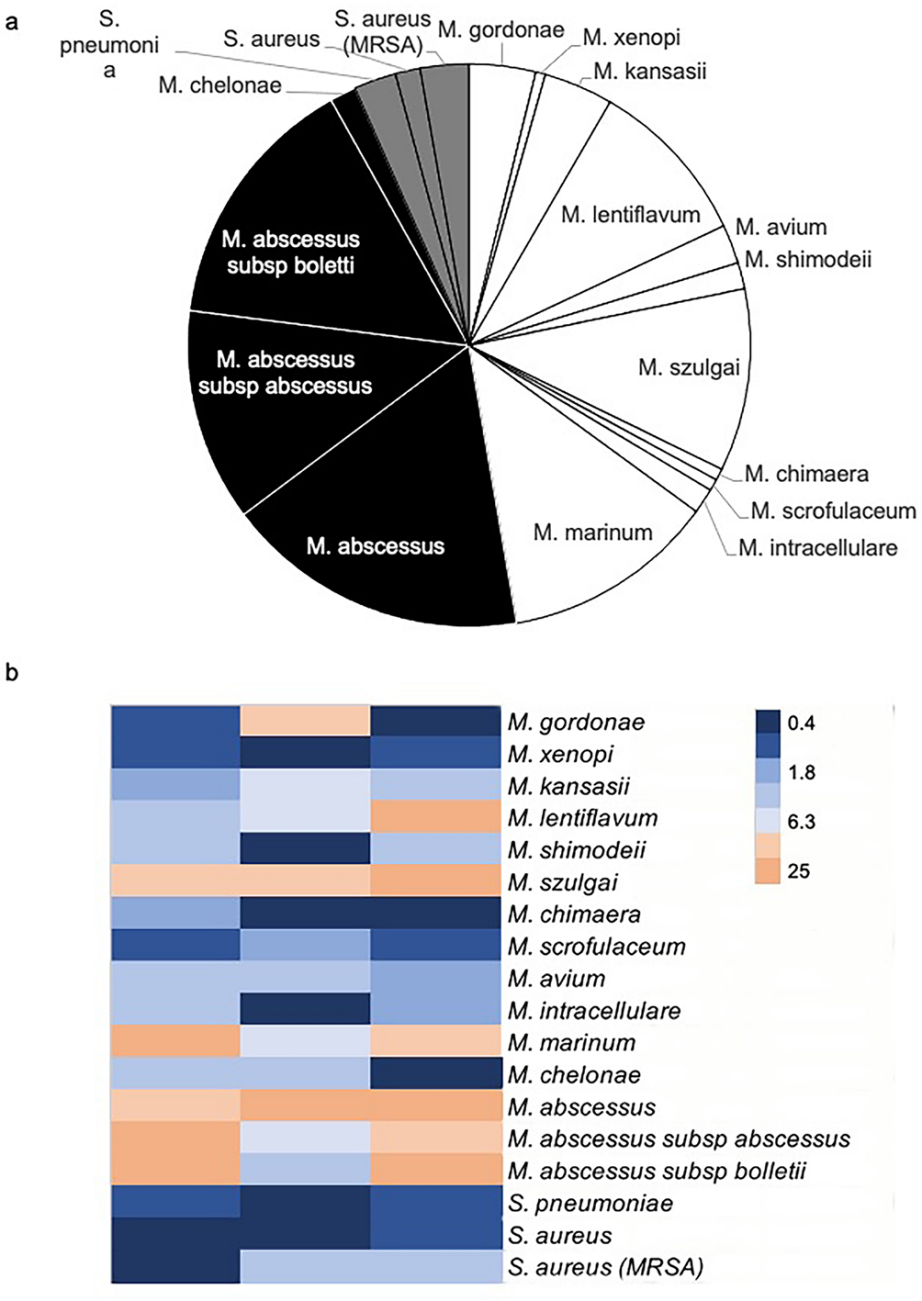
Antimicrobial activity of NZX. (**a**) NZXs impact on fast growing and slow growing bacteria. NZX showed potent activity on both fast growing (black) and slow growing (white) clinical NTM isolates, and clinical Gram-positive isolates (grey). We could not observe any clear-cut differences in MIC_99_ values between slow growing strains and fast growing NTM strains. The pie-chart is showing the concentrations of NZX needed to kill the different bacteria. (**b**) Heatmap showing the unique distribution profiles of resistance to NZX (MIC_99_) on three clinical isolates of each bacteria.

This peptide was previously shown to kill both drug sensitive and MDR clinical isolates of *M. tuberculosis*^9^. When assayed for antimicrobial activity against a broad spectrum of mycobacteria, NZX showed potent activity against several clinical NTM isolates and three clinical Gram-positive isolates, namely *S. aureus*, MRSA and *S. pneumoniae* (Fig. 1a,b, Table S1). The selected NTM isolates required higher inhibitory NZX concentrations, i.e. *M. abscessus subsp.*, *M. szulgai, M. lentiflavum,* and *M. marinum.* In contrast, NZX did not possess any activity against the Gram-negative *Escherichia coli* (MIC > 100 uM). We could not observe any clear-cut differences in MIC values between slow growing strains and fast growing NTM strains (Fig. 1a,b).

### Interaction of the peptide with current TB drugs

The interaction between NZX and rifampicin, isoniazid, ethambutol or amikacin were analysed by checkerboard assay and MTT (Table 1, Fig. S1a,b). Combination treatment suggested that NZX interactions with TB-drugs displayed mostly additive or indifferent interaction scores. None of the drug combinations displayed synergy or possessed an antagonistic effect. Interestingly, NZX had an additive effect when combined with EMB and INH.

**Table 1:** FIC index of interactions between the peptide and first- and second-line anti-TB drugs.

Drug combinations^a^	FIC index^b^	Interpretation
NZX: RIF	2.1	Indifference
NZX: EMB	0.75	Additive
NZX: INH	1	Additive
NZX: AMK	1.1	Indifference

^a^NZX (peptide), RIF (rifampicin), INH (isoniazid), EMB (ethambutol), AMK (amikacin).

^b^FIC Index borders: synergistic (≤ 0.5), additive (> 0.5–1), indifference (1–4), antagonistic (> 4).

### Development of resistance to the peptide

To examine the potential for the development of resistance against NZX, we attempted to isolate spontaneous mutants of *M. smegmatis* with reduced drug susceptibility. Bacteria were grown in several independent cultures without NZX and subsequently exposed to drug at different concentrations on agar plates or in liquid cultures. We did not detect any resistant mutants irrespective of drug concentration or selection condition used.

### Evaluation of the drug additive effect in a murine infection model

**Figure 2:**
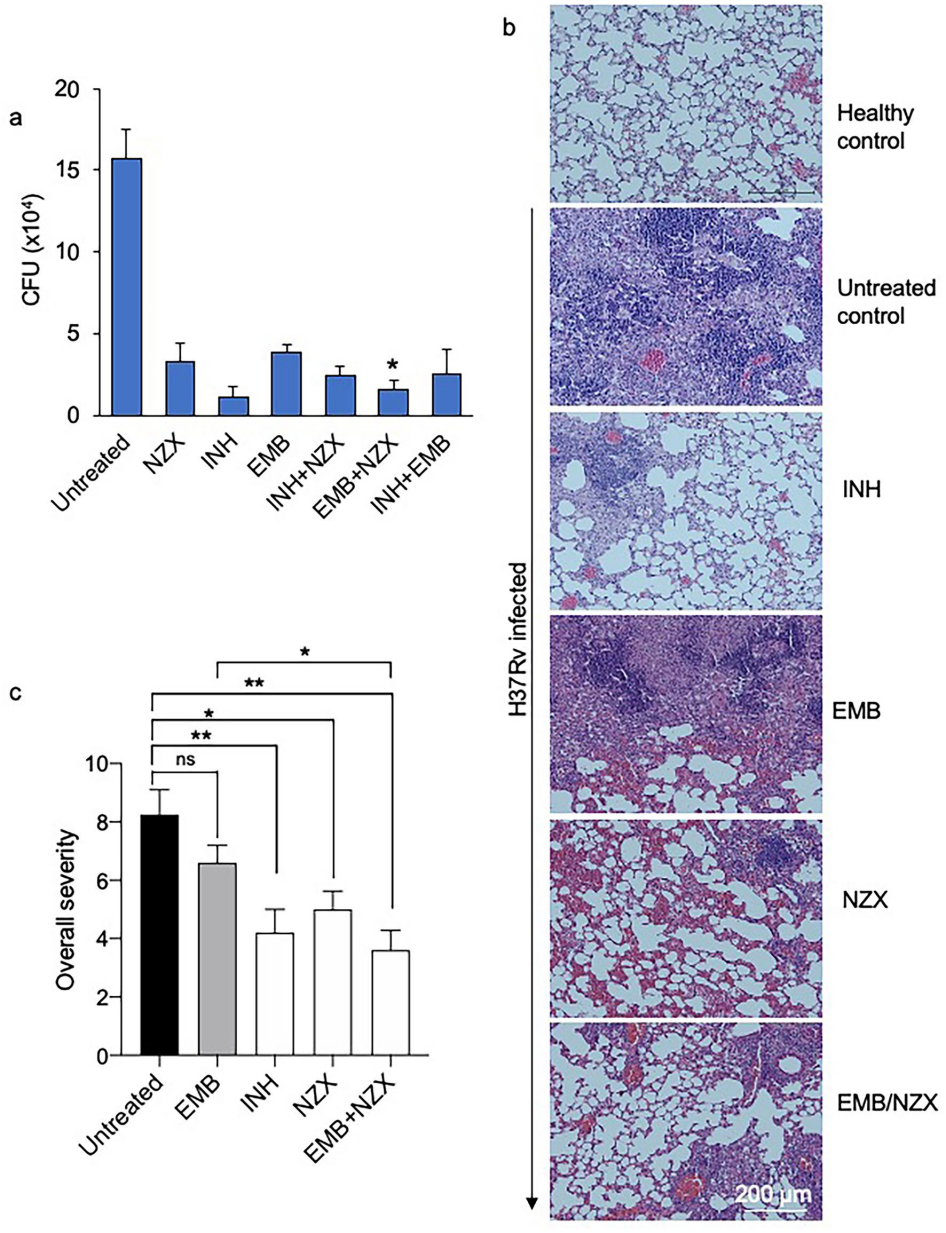
Murine evaluation of drug combinations. (**a**) Treatment induced bacterial reduction compared to the untreated control (isoniazid p = 0.0159, ethambutol p = 0.0159, NZX p = 0.0159). Combination treatment with ethambutol/NZX reduced bacterial load significantly more than ethambutol treatment alone (p = 0.0317), but not from NZX treatment alone (p = 0.4206). We found no significant difference between the other groups. Results presented as mean ± sd. All *p* values were calculated by unpaired Student’s *t*-test, Mann–Whitney or ANOVA (*p < 0.05). (**b**) Representative eosin (H&E) staining showing lung sections from *M. tuberculosis* H37Rv infected mice and uninfected control mice. H&E staining of untreated lungs showed tissue destruction and granuloma formation. Mice treated for four weeks with INH, NZX or NZX/EMB showed decreased tissue destruction. Scale bar 200 μm. (**c**) Results of the blinded lung inflammatory score. Data are presented as the mean ± SEM (*n* = 4–5). *p* values were determined using a one-way ANOVA with Tukey’s post hoc test. *p ≤ 0.05; **p ≤ 0.01.

To confirm our results from the drug interaction experiments, we performed a murine TB infection experiment with *M. tuberculosis* H37Rv (Fig. S2). The mean bacterial implantation dose in the lungs, measured two days after infection, was 677 CFU/ml. The bacterial load was 4.46 × 10^5^ after 28 days when the treatment started and 1.57 × 10^5^ at the end of the experiment. Treated animals received three doses/week of NZX, isoniazid and/or ethambutol for four weeks. All untreated mice survived the duration of the experiment. In the treated groups, we observed a general CFU reduction of 82% compared to untreated mice (p = 0.0019) (Fig. 2a). Bacterial reduction in the isoniazid group compared to the untreated control was 92% (p = 0.0159); for ethambutol and NZX groups it was 79% (p = 0.0159) and 75% (p = 0.0159) respectively. We found no significant difference between isoniazid and NZX (p = 0.1508), or between NZX treatment and ethambutol treatment (p = 0.6905). Comparing the peptide/drug combinations, we found that combination treatment with ethambutol/NZX reduced bacterial load significantly more than ethambutol treatment alone (p = 0.0317), but not from NZX treatment alone (p = 0.4206). The combination of isoniazid/NZX was not different from the individual drugs (p = 0.0873 and p = 0.5000 respectively).

### NZX attenuates inflammation during chronic tuberculosis

Analysis of lung inflammatory criteria showed that H37Rv infected mice treated with the NZX/ethambutol combination had the least tissue destruction (i.e. perivascular infiltration, peribronchiolar infiltration and alveolar infiltration) (Fig. 2b,c). Of the investigated drugs, isoniazid preserved alveolar structure best, while ethambutol treated mice had more severe tissue destruction. The INH/NZX scores were identical to INH scores, while INH/EMB scored similar to NZX scores (data not shown).

### Serum half-life of NZX

**Figure 3:**
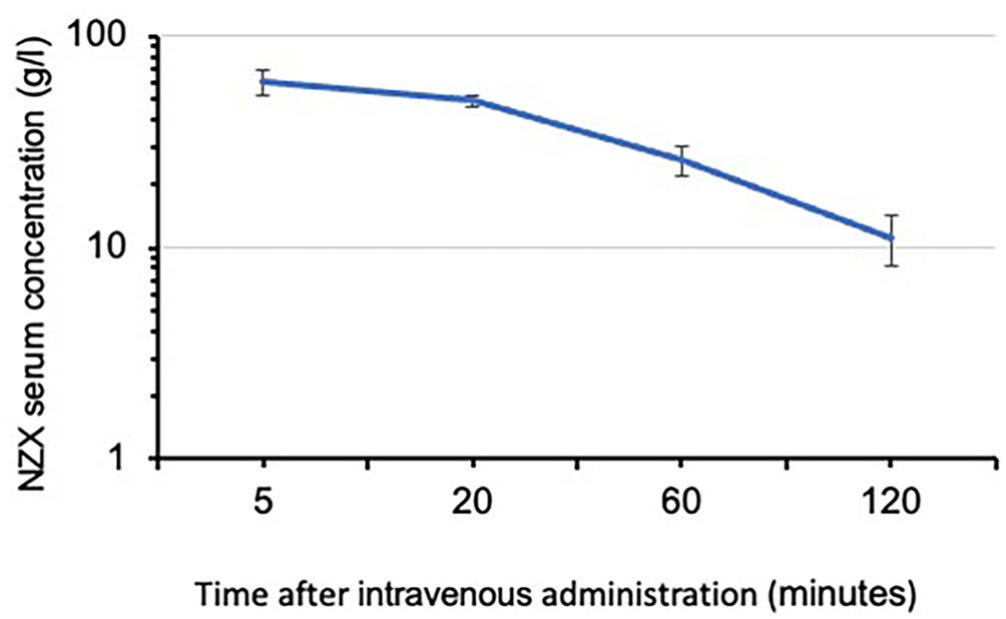
Pharmacokinetic studies of NZX. Mice received a single dose of NZX 33 mg/kg through intra-venous route (n = 3 at each timepoint). The maximum observed mean concentrations for intravenous dosing was 61 mg/l after 5 min and the terminal half-life was estimated to 42 (± 5.6) min. After 240 min the NZX was not detected in the circulation.

We analysed the pharmacokinetics of the peptide by measuring serum terminal half-life, as the time required for the serum concentration of a drug to decrease by 50%^11^. Previous studies revealed that plectasin had a terminal serum elimination half-life of 51 min^8^. We measured the pharmacokinetic behaviour of NZX in vivo by administering mice with a single intravenous dose of 33 mg/ kg NZX. We determined the maximum observed mean concentration for intravenous dosing of NZX to be 61 mg/l after 5 min and the terminal half-life was estimated to 42 min (Fig. 3). To further investigate the impact of serum we analysed the function of NZX as MIC_99_. We observed stable and similar MIC values to those previously reported in Tenland et al.^9^ after incubation for up to three hours.

## Discussion

The endless spread of antibiotic resistance is driving the quest for novel drugs. CHDPs, until recently mostly for topical applications, are now at different stages of clinical trials. Previously, NZX was shown to possess bactericidal activity against *M. tuberculosis* in vitro and able to reduce the bacterial load in a murine infection model to levels comparable to rifampicin alone^9^. In this study, we found that NZX displayed an additive effect when combined with ethambutol and isoniazid. Interpretation of experimental drug combinations studies is known to be fraught with difficulties but nevertheless provides a starting point towards possible clinical use^12^. As confirmation we tested the combination of drugs in a chronic murine TB infection model, where a thrice-weekly regimen confirmed the additive effect with ethambutol after 4 weeks. As in previous studies, we compared NZX activity to antibiotics routinely used to treat tuberculosis as this comparison provides us with a better understanding of the potential of NZX. Tissue analysis also revealed that NZX treated mice exhibited better preserved lung structure compared to ethambutol alone treated animals. This observation is interesting and could be possibly explained by better NZX tissue penetration^9^. Ethambutol only exhibits modest activity against *M. tuberculosis* experimentally, but it was chosen to be a part of the first-line chemotherapy based on its clinical performance^13^. This drug is also used as part of multidrug regimens to treat NTM infections, although it has been associated with tissue damage^14–16^. In our murine studies, although three times as much ethambutol was administered compared to NZX, the peptide managed to preserve lung tissues even in combination with ethambutol.

When assayed for antimicrobial activity against a broad range of other mycobacteria and Gram-positive bacteria as well as *M. tuberculosis*, NZX showed potent activity against several other clinical isolates. Among these were MRSA and *S. pneumoniae*, while the Gram-negative *Escherichia coli* was not affected. NTMs are mostly facultative pathogens capable of causing chronic granulomatous diseases that can be pathologically indistinguishable from TB. The frequency of NTM isolation from respiratory samples and NTM pulmonary disease is increasing in many countries^17^. Drug development for environmental NTMs is challenging as these mycobacteria have developed elaborate mechanisms to achieve resistance and have the ability to switch between antibiotic-susceptible and resistant variants^18^. Of the hitherto 198 known NTM species, *M. avium, M. intracellulare, M. chimaera, M. kansasii*, and *M. abscessus* are the species most commonly reported to cause human disease. *M. abscessus* accounts for the majority of lung disease caused by rapidly-growing mycobacteria and is difficult to treat due to high level of innate resistance^19^. The common predisposing conditions for *M. abscessus* infection are pulmonary diseases such as bronchiectasis, COPD and cystic fibrosis, where the increased mucus production pose additional problems for treatment. Through analysis of bactericidal concentrations of the peptide against these strains, we found differences between the slow growing *M. avium, M. intracellulare, M. chimaera, M. kansasii* and the rapidly growing *M. abscessus* subsp. which required higher concentrations of the peptide. The observed differences in bactericidal concentrations for different mycobacterial strains is likely due to their varied drug susceptibility^18, 20^. Compared to these studies we observed that the susceptibility of NTMs to NZX is constant.

The possibility of intravenous therapy is important in patients with severe TB. We demonstrated that NZX has a serum terminal half-life similar to plectasin^21–23^. To examine the potential for the development of resistance against NZX, we attempted to isolate spontaneous mutants of *M. smegmatis* with reduced peptide susceptibility. We did not isolate any resistant mutants irrespective of drug concentration or selection condition used, suggesting that if resistant mutants do exist, they emerge at a frequency of < 10^–9^. For other peptides (e.g. PR-39, colistin, protamine), we have previously isolated resistant mutants at frequencies of approximately 10^–7^ to 10^–6^/cell/generation^24^. Thus, in comparison to many other peptides, as well as antibiotics, NZX appears to have a low potential to develop resistance.

To summarize, we demonstrate that NZX has an additive effect with ethambutol in a murine TB infection model. We note that NZX exhibits significant antimicrobial capacity when assayed against a broad range of clinical isolates including NTMs and Gram-positive bacteria. No antagonism between NZX and standard TB drugs was observed, further supporting the therapeutic potential of this peptide.

## Methods

### Peptide

The peptide was manufactured by solid phase peptide synthesis, followed by cyclisation of three natural occurring di-sulphide bonds and purification by sequential chromatography steps (PolyPeptide Laboratories AB, Limhamn, Sweden). The purity (> 97%) of the peptide was confirmed by high-performance liquid chromatography^9^.

### Bacteria

For screening experiments, 15 clinical NTM isolates (*M. avium*, *M. abscessus*, *M. abscessus* subsp. *boleti*, *M. abscessus* subsp. *abscessus*, *M. marinum*, *M. chelonae*, *M. intracelluare*, *M. kansasii*, *M. lentiflavum*, *M. scrofulaceum*, *M gordonae*, *M. szulgai*, *M. shimodeii*, *M. xenopi*, and *M. chimaera*, as well as five clinical isolates of the Gram positives S*taphylococcus aureus* (*S. aureus*), Methicillin-resistant *S. aureus* (MRSA) and *Streptococcus pneumoniae* (*S. pneumoniae*) were obtained from Clinical Microbiology, Regional Laboratories Skåne, Lund, Sweden. *Mycobacterium bovis* Bacillus Calmette-Guerin (BCG) Montreal strain containing the pSMT1-*luxAB* plasmid^25^ was a kind gift from Dr. Brian Robertson, Imperial College London, UK. *Mycobacterium smegmatis* mc^2^155 (a kind gift from Prof. Leif Kirsebom, Department of Cell and Molecular Biology, Box 596, Biomedical Centre, Uppsala, Sweden) was used for antibiotic resistance screening experiments. For murine TB infection experiments, we used *M. tuberculosis* H37Rv (a kind gift from Christophe Guilhot, Institut de Pharmacologie et de Biologie Structurale (IPBS), Toulouse, France)^9^.

Briefly, mycobacterial strains were grown in Middlebrook 7H9 culture medium, supplemented with 10% ADC enrichment (Becton Dickinson, Oxford, UK)^9^. For BCG hygromycin (50 mg/l; Roche, Lewes, UK) was added to the broth. *M. tuberculosis* H37Rv was cultured to mid-log phase in Middlebrook 7H9 culture medium, supplemented with 0.05% Tween 80, 0.2% glycerol and 10% oleic acid-albumin-dextrose-catalase (OADC) enrichment (Becton Dickinson, Oxford, UK). Mycobacterial cultures were then washed twice with sterile PBS and re-suspended in broth and dispensed into vials. Glycerol was added to a final concentration of 50% and the vials were frozen at − 80 °C. Prior to each experiment, a vial was defrosted, added to 19 ml of 7H9/ADC/hygromycin medium, and incubated with shaking for 72 h at 37 °C, except *M. marinum* that was grown at 28 °C. Mycobacteria were then centrifuged for 10 min at 3000×*g*, washed twice with PBS, and re-suspended in 10 ml of PBS.

Clinical strains of *Staphylococcus aureus* and *Streptococcus pneumoniae* were cultured from Blood Agar plates in LB. Both strains were cultured overnight with shaking at 37 °C.

*Escherichia coli* 1177 of serotype O1:K1:H7, was isolated from urine of a child with acute pyelonephritis in a previous clinical study by Mårild et al.^26^. The strain was maintained in deep agar stabs sealed with sterile paraffin, passaged on tryptic soy agar, grown overnight in static Luria broth and harvested by centrifugation at 1500×*g* for 10 min. The pellet was resuspended in 0.01 M of PBS (pH 7.2) to a concentration of 1–2 × 10^9^ CFU/ml.

The optical density of bacterial suspension was measured at 600 nm (SmartSpecTM Plus, BIO-RAD) before experiments, and adjusted to an OD of 0.01 (~ 10^6^ CFU/ml).

### Isolation of *M. smegmatis* mutants with reduced susceptibility to the peptide on solid medium

NZX mycobacterial MIC (minimal inhibitory concentration) values were investigated in our previous study^9^. Bacteria were plated on duplicate agar plates supplemented with 12.6 µM (2 × MIC) to 100.8 µM (16 × MIC) of peptide in twofold increments. For each NZX concentration, 2 × 100 µl of stationary cell culture (approximately 2 × 10^8^ cells per NZX concentration) was spread on the plates which were incubated for 48 h at 37 °C. Colonies with reduced susceptibility were picked with a loop and grown in 200 µl of 12.6 µM (2X MIC) NZX for 48 h at 37 °C shaking (110 rpm). From the wells with growth, cells were spread with a loop on agar plates without peptide for isolation of single colonies. Single colonies were analyzed for MIC.

### Isolation of mycobacterial mutants with reduced susceptibility to the peptide in liquid culture

Triplicate samples of 100 µl bacterial culture were added to a 96 well plate supplemented with NZX peptide concentrations ranging from 12.6 µM (2 × MIC) to 100.8 µM (16 × MIC) in twofold increments. Each well contained 100 µl of medium containing peptide plus 100 µl of bacterial cell suspension (10^8^ cells). The 96-well plate was incubated for 24 h at 37 °C shaking (110 rpm) and then 100 µl culture from each of the triplicate wells was spread on plates without peptide and colonies were isolated. A total of 23 colonies were isolated from the 8× and 16× MIC plates and analyzed in a MIC assay.

### Minimum inhibitory concentration (MIC)

Resazurin microtiter assays (REMA) were used to determine the minimum inhibitory concentration (MIC_99_) for NZX against the strains mentioned above^27^. NZX (10 µl) was added to bacterial suspensions (90 µl) on a 96-well plate at a concentration range between 25.0–0.2 µM. A negative control, positive control and two extra controls were diluted from OD 0.01 to 1/10 and 1/100 and were included on the plate and incubated at 37 °C, 5% CO_2_ (Fig. 1). MIC was determined by the color change using resazurin (1:10 v/v, PrestoBlue Cell viability reagent, Thermo Scientific). MIC was determined after one week for most strains by adding 10 µl resazurin followed by incubation overnight^27^, corresponding to 99% inhibition.

### NZX interactions with current TB antibiotics

Drug interactions between NZX and the current TB drugs rifampicin (RIF), isoniazid (INH), ethambutol (EMB) and amikacin (AMK) were analyzed with checkerboard assay using resazurin and MTT. Bacterial suspensions (80 µl) were exposed to each drug (10 µl; RIF (0.002–0.06 µg/ml), EMB (0.06–4 µg/ml), INH (0.03–2 µg/ml), AMK (0.02–1 µg/ml) with NZX (0.88–110 µg/ml) to test for synergy on a 96-well plate. After drug exposure, resazurin or MTT were used to quantify living cells. The resazurin (10 µl (1:10 v/v, PrestoBlue Cell viability reagent, Thermo Scientific) was added to the plate and incubated at 37 °C overnight. A color change from blue to pink indicated living cells. Similarly, for plates being analyzed with MTT (1:10 v/v, Sigma), 10ul was added, incubated for one hour at 37 °C, whereafter the cells were lysed with DMSO. Absorbance was read at 500 nm with a Tecan Infinite F200 reader. Growth inhibition corresponded to absorbance recorded for 1:100 control well (99% inhibition).

The MICs of individual drugs and combination therapies were used to calculate the fractional inhibitory concentration (FIC) index. FIC index score was calculated with the following equation, ΣFIC = FIC_A_ + FIC_B_ = (C_A_/MIC_A_) + (C_B_/MIC_B_), where MIC_A_ and MIC_B_ are the MICs of drugs A and B alone, and C_A_ and C_B_ are the concentrations of the drugs in combination, in representative wells wherein no growth is recorded. The obtained FIC index scores represent different types of interactions; < 0.5 is synergy, 0.5–1 is additive, 1–4 is indifference and > 4 is antagonistic.

A negative control, a positive control and two extra controls diluted from OD 0.01 to 1/10 and 1/100 were included on the plate and incubated at 37 °C, 5% CO_2_, representing 90% and 99% growth inhibition (Supplementary Fig. S1). The plate also contained an independent row of individual drug along the x and y-axis, representing MIC values for the respective drugs. MIC was determined for resazurin (1:10 v/v, PrestoBlue Cell viability reagent, Thermo Scientific) by transferring 10 µl followed by overnight incubation. For the MTT assay (1:10 v/v, Sigma) 10 ul of the MTT labelling reagent was added with incubation for one hour at 37 °C, whereafter the cells were lysed with DMSO. Absorbance was read at 500 nm with a Tecan Infinite F200 reader.

### NZX pharmacokinetics

Animal experiments were performed according to the Swedish Animal Welfare Act SFS 1988:534 in compliance with the ARRIVE guidelines and were approved by the Animal Ethics Committee of Malmö/Lund, Sweden. Male BALB/c mice, aged 8–10 weeks, were obtained from Jackson Laboratories (Bar Harbor, ME, USA) and maintained under standard 12:12 dark light cycle with food and water ad libitum. They were maintained in the animal facility at the Department of Microbiology, Immunology, and Glycobiology, Lund University, Lund, Sweden. They were anaesthetized by isoflurane inhalation for 10–20 s. NZX (33 mg/kg) was administered intravenously (i.v.) via the lateral tail vein in 12 (BALB/c) mice. Three mice were culled after 5, 20, 60 and 120 min, and their blood collected on dry ice. The clotted blood samples were centrifuged, and serum collected.

Quantification of NZX in serum was done by LC/MS analysis. A dilution series of known concentrations of NZX were prepared in mouse serum (M5905, SIGMA). Standard additions of the analyte NZX were performed in serum taken at time point 240 min before sample preparation allowing for the control of recovery and quantification. Duplicates samples of 25 µl of serum, recovery controls and standards were precipitated by the addition of 75 µl 10% TFA in 20% AcCN. The samples and standards were then kept at 4 °C for 30 min before centrifugation at 20,817 rcf, 4 °C for 20 min. The supernatants from time points 5 and 20 min were diluted 50 times by 0.1% FA in 50% ACN (acetonitrile), and the 60 min time points were diluted 20 times. The 240 min time points and standards were not further diluted before analysis by LC/MS (Acquity UPLC i-class/Xevo G2-S QTOF, Waters). The column used for separation was a C18 column (Acquitiy UPLC HSS T3 1.8 µm) eluted by a 5 min gradient from 80% eluent A (0.02% TFA, 0.08% FA) to 95% eluent B (0.02% TFA, 0.08% FA in ACN) at a flow rate of 0.3 ml/min. The NZX was quantified against the external standard curve prepared in mouse serum and the concentrations were corrected by the determined recovery.

For the human serum stability assay, the peptide was incubated in human serum for 1, 2 and 3 h at 37 °C. The serum was used to prepare serial dilutions of the peptide. After each time point, 10 μl of serum-incubated peptide was added to 90 μl BCG suspension and incubated at 37 °C for between 4 and 7 days before PrestoBlue was added, and analysed in a spectrophotometer at 580 nm.

### Murine treatment model

Animal procedures were performed under a license issued by the UK Home Office and in accordance with the Animal Scientific Procedures Act of 1986, and in compliance with the ARRIVE guidelines. Six to eight week old female BALB/c mice (Charles River Ltd, UK) were maintained in biosafety containment level 3 (BSL3) facilities at Imperial College London, randomised in groups of 5 in Tecniplast HEPA-filtered cages according to institutional protocols with food and water ad libitum^9^. Mice were infected with ~ 7 × 10^3^ CFU/ml of *M. tuberculosis* H37Rv in 35 µl via the intranasal route under gaseous anaesthesia (control group, n = 8—including 3 mice to check bacterial implantation in the lungs on day 2**—**and groups receiving the two first-line drugs isoniazid (INH) or ethambutol (EMB), or NZX, alone or in combinations). Following 28 days of infection, 5 control mice were culled by cervical dislocation to determine the bacterial burden before treatment, while 7 groups were treated 3 times per week for 4 weeks with 25 mg/kg isoniazid, 100 mg/kg ethambutol, or 33 mg/kg NZX alone or in combinations diluted in 35 μl PBS by intranasal administration. The control group received 35 μl PBS by the same route. Following treatment, mice were culled, and the lungs were aseptically removed. The left lobe of the lung was placed in 10% buffered formalin for 24 h, for histology. The remaining tissue**s** were homogenized in PBS containing 0.05% Tween-80, serially diluted and plated on Middlebrook 7H11 agar plates supplemented with 0.5% glycerol and 10% OADC. The number of colony forming units (CFU) from all mice was enumerated 21 days later.

### Histology

Formalin fixed tissue was transferred to 70% ethanol then embedded in paraffin blocks. For the histopathological analysis, 5 μm sections were stained with hematoxylin and eosin as described previously^9^. Sections were scored blind for three inflammatory criteria (perivascular infiltration, peribronchiolar infiltration, and alveolar infiltration), and scored semi quantitatively on a scale of 0–4 (0, normal; 1, minimal; 2, mild; 3, moderate; 4, marked). An overall severity score was calculated for each animal by adding the individual scores, and scores for animals from each experimental group were pooled and averaged. Slides were examined by fluorescence microscopy (AX60, Olympus Optical). Richard-Allan Scientific Signature Series Hematoxylin 7211 and Eosin-Y 7111 (Thermo Scientific) was used to counterstain the tissue sections.

### Ethics statement

All animal procedures were reviewed by the Imperial College Animal Welfare and Ethical Review Board and performed under licences issued by the UK Home Office in accordance with the Animal Scientific Procedures Act of 1986 (70/7160 and 70/8653). The Animal Ethical Review Board (Malmö/Lund district) approved the control animal studies (Dnr M 7–15). The Local Ethical Review Board Dnr 2011/403 and 2014/35 approved the donation of blood from adult human volunteers for the in vitro studies (Lund district). The healthy volunteers were provided with verbal and written information about the study’s purpose, duration, potential risks and benefits, informed consent was provided by signing a document approved by the Local Ethical Review Board. The blood was pooled. All methods in this manuscript were performed in accordance with the relevant guidelines and regulations.

### Statistical analysis

Graphs and statistics were generated using the Prism software (version 6.1). Significance, where indicated, was calculated using the unpaired Student’s *t*-test or ANOVA. For the murine experiment comparing seven groups; untreated and treated with NZX, isoniazid and ethambutol alone or in combinations, we performed ANOVA followed by Dunnett’s multiple comparison and Mann–Whitney between groups. Significance was accepted at *p < 0.05, **p < 0.01, or ***p < 0.001.

## Supplementary Information

Supplementary Information.
