# Supplementary material for: A broad spectrum anti-bacterial peptide with an adjunct potential for tuberculosis chemotherapy
Source: Sci Rep. 2021 Feb 18;11:4201. doi: 10.1038/s41598-021-83755-3 (PMC7892554; doi:10.1038/s41598-021-83755-3)

A broad spectrum anti-bacterial peptide with an adjunct potential for tuberculosis chemotherapy

Komal Umashankar Rao, Domhnall Iain Henderson, Nitya Krishnan, Manoj Puthia, Izabela Glegola-Madejska, Lena Brive, Fanny Bjarnemark, Anna Millqvist Fureby, Karin Hjort, Dan I. Andersson, Erik Tenland, Erik Sturegård, Brian D. Robertson, Gabriela Godaly

Supplementary Table

Table S1. Antimicrobial activity of the peptide.

Supplementary Figures

Figure S1. Representative set-up for REMA and checkerboard assays. (**a**) MIC_{99} values for NTMs evaluated from REMA assay (**b**) Combination therapy was evaluated using checkerboard assay. Two drugs were added to wells containing BCG. Drug concentration followed a decreasing order in their respective x and y axis. FIC index was calculated for the appropriate well indicated in the Figure.

Figure S2. Schematic representation of experimental setup for murine pulmonary TB with *M. tuberculosis* H37Rv. Mice were infected with ~$7x10^3$ CFU/ml of *M. tuberculosis* H37Rv via the intranasal route. Following 28 days of infection, groups were treated three times per week during for four weeks with isoniazid (INH), ethambutol (EMB), or NZX alone or in combinations by intranasal administration.

Supplementary Table 1

		Minimal inhibitory concentration (mg/l)						
Species	No of isolates tested	0.4	0.8	1.6	3.2	6.3	12.5	25
M. gordonae	3	1	1				1	
M. xenopi	3	1	2					
M. kansasii	3			1	1	1		
M. lentiflavum	3				1	1		1
M. avium	3			1	2			
M. shimodeii	3	1		1	1			
M. szulgai	3						2	1
M. chimaera	3	2		1				
M. scrofulaceum	3		2	1				
M. intracellulare	3	1		1	1			
M. marinum	3					1	1	1
M. abscessus	3						1	2
M. abscessus subsp abscessus	3					1	1	1
M. abscessus subsp boletti	3				1			2
M. chelonae	3	1			2			
S. pneumonia	5	1	2			2		
S. aureus	5	2	2		1			
MRSA	5		1		3	1		
*E. coli**	3							

* MIC >100 uM

Supplementary Figure 1

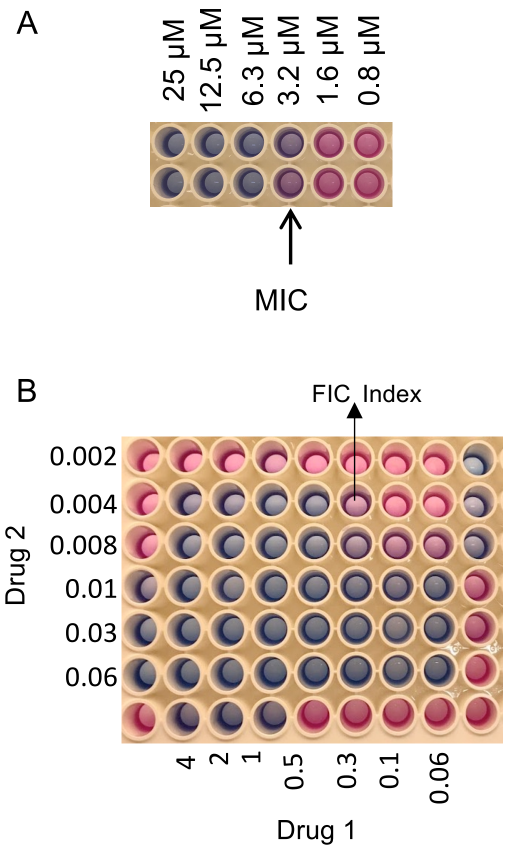

Supplementary Figure 2

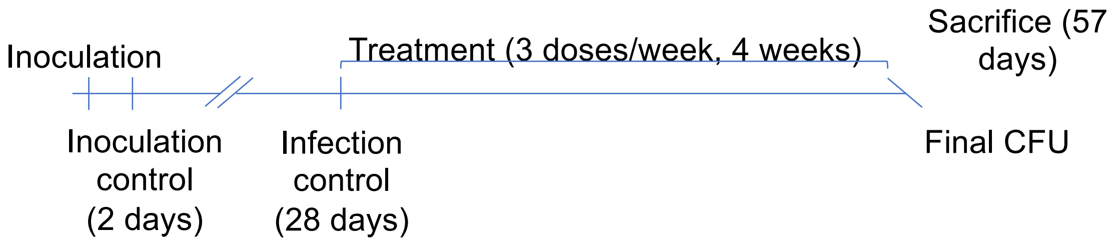

Supplement: Supplementary file 1 — Supplementary Information. [file 41598_2021_83755_MOESM1_ESM.pdf]
